# The Louisville Embroglio

**Published:** 1877-01-20

**Authors:** 


### THE LOUISVILLE EMBROGLIO.

We have received, through the press and otherwise, sundry articles bearing upon the unfortunate controversy among medical brethren in Louisville. At present, we have not space to devote to the subject, further than to express a hope that the truth and the right will prevail, no matter whom it affects. Good men throughout the country, though having no voice in settling local controversies, always regard with contempt the efforts of those who seek to put down the truth and give supremacy to error. No honor will attach to the party who gains a point by the use of unfair and dishonorable means. No one can be considered honorable who will indorse an act committed by his friends and condemn the same act when committed by his enemies.

